# Comparative *in silico *analysis identifies *bona fide *MyoD binding sites within the Myocyte Stress 1 gene promoter

**DOI:** 10.1186/1471-2199-9-50

**Published:** 2008-05-19

**Authors:** Samir Ounzain, Caroline S Dacwag, Nilesh J Samani, Anthony N Imbalzano, Nelson W Chong

**Affiliations:** 1 Cardiology Group, Department of Cardiovascular Sciences, University of Leicester, Clinical Sciences Wing, Glenfield General Hospital, Leicester, LE3 9QP, UK; 2 Department of Cell Biology, University of Massachusetts Medical School, 55 Lake Avenue North, Worcester, MA 01655, USA

## Abstract

**Background:**

Myocyte stress 1 (MS1) is a striated muscle actin binding protein required for the muscle specific activity of the evolutionary ancient myocardin related transcription factor (MRTF)/serum response factor (SRF) transcriptional pathway. To date, little is known about the molecular mechanisms that govern skeletal muscle specific expression of MS1. Such mechanisms are likely to play a major role in modulating SRF activity and therefore muscle determination, differentiation and regeneration. In this study we employed a comparative *in silico *analysis coupled with an experimental promoter characterisation to delineate these mechanisms.

**Results:**

Analysis of MS1 expression in differentiating C2C12 muscle cells demonstrated a temporal differentiation dependent up-regulation in *ms1 *mRNA. An *in silico *comparative sequence analysis identified two conserved putative myogenic regulatory domains within the proximal 1.5 kbp of 5' upstream sequence. Co-transfecting C2C12 myoblasts with *ms1 *promoter/luciferase reporters and myogenic regulatory factor (MRF) over-expression plasmids revealed specific sensitivity of the *ms1 *promoter to MyoD. Subsequent mutagenesis and EMSA analysis demonstrated specific targeting of MyoD at two distinct E-Boxes (E1 and E2) within identified evolutionary conserved regions (ECRs, α and β). Chromatin immunoprecipitation (ChIP) analysis indicates that co-ordinated binding of MyoD at E-Boxes located within ECRs α and β correlates with the temporal induction in *ms1 *mRNA.

**Conclusion:**

These findings suggest that the tissue specific and differentiation dependent up-regulation in *ms1 *mRNA is mediated by temporal binding of MyoD at distinct evolutionary conserved E-Boxes within the *ms1 *5' upstream sequence. We believe, through its activation of *ms1*, this is the first study to demonstrate a direct link between MyoD activity and SRF transcriptional signalling, with clear implications for the understanding of muscle determination, differentiation and regeneration.

## Background

During mammalian embryogenesis, the development of skeletal muscle is mediated by a co-ordinated series of events that begins with commitment of mesodermal precursor cells to the skeletal muscle lineage, followed by myoblast fusion and the subsequent progression of a programme of muscle specific gene expression [1-3]. A specialised group of transcription factors control this process of myogenic specification and differentiation. These factors, designated the myogenic regulatory factors (MRFs), include four basic helix-loop-helix (bHLH) E-Box binding proteins: MyoD, Myf5, Myogenin and MRF4 [4]. During development MyoD and Myf5 dictate myoblast specification while Myogenin and MRF4 regulate terminal differentiation [5,6]. In collaboration with the MRFs, the MADS-box myocyte enhancer factor (MEF) family of proteins contribute to the programme of muscle specific gene expression [7,8].

Serum response factor (SRF), a MADS box transcription factor related to the MEFs, also regulates skeletal muscle gene expression through binding of a DNA sequence known as the serum response element (SRE) or CArG box [9-11]. In addition to binding and regulating numerous muscle specific promoters [12,13], perturbation of SRF activity severely impairs myoblast fusion and differentiation [14-16]. Confirming an important role for myogenic SRF activity, a conditional skeletal muscle specific knockout of SRF results in severe skeletal muscle myopathy that results in perinatal lethality [17].

SRF activity is dependent on its interaction with a range of cell-type specific and signal responsive co-factors [18]. Myocardin, the founding member of a family of extraordinarily powerful myogenic SRF co-activators [19], has been shown to be necessary and sufficient for cardiac and smooth muscle specific gene expression [9,20,21]. Unlike myocardin, the myocardin-related transcription factors (MRTFs), MRTF-A (also known as MAL/MKL1/BASC) and MRTF-B (MKL2), are expressed in skeletal muscle in addition to multiple other cell types [22-24].

A requisite role for the MRTFs in skeletal muscle development has been inferred from experiments in cultured muscle cells, in which RNAi mediated knock-down of MRTF-A repressed SRF-dependent gene expression resulting in impaired myoblast fusion and subsequent formation of multinucleated myotubes [25]. Transgenic mice expressing a dominant-negative form of MRTF-A displayed a phenotype reminiscent of the skeletal muscle SRF knock out mice supporting an important role for the MRTFs in the control of muscle fiber growth and maturation [17]. In contrast to myocardin, which is constitutively nuclear, the MRTFs shuttle between the cytoplasm and the nucleus with nuclear accumulation required for SRF trans-activation. Muscle specific mechanisms, which promote MRTF nuclear accumulation, represent important regulatory pathways in the process of myogenic differentiation via the MRTF/SRF signalling axis [9].

We, and others, have previously identified a novel striated muscle specific actin binding protein, myocyte stress 1 (MS1, also known as STARS) [26,27] which has the ability to synergistically activate SRF-dependent transcription through a Rho-A dependent mechanism. Kuwahara and colleagues subsequently demonstrated that STARS (mouse homologue of MS1) activates SRF dependent transcription by inducing the nuclear accumulation of MRTF-A and -B through a Rho-A dependent mechanism [28]. STARS perturbation via RNAi resulted in a significant attenuation of muscle specific SRF activity suggesting that endogenous STARS is an important component of the muscle specific MRTF/SRF transcriptional pathway [28]. In support of this we have shown that ectopic MS1 expression results in an increased expression of characterised MRTF/SRF target genes (Koekemoer AL and Chong NW, unpublished) [29]. Interestingly, we have recently shown that morpholino knockdown of zebrafish *ms1 *(*zms1*) resulted in severe musculoskeletal deformities with curvature and shortening of the longitudinal axis [30]. This data supports the previous studies and demonstrates that MS1 is a central component of the evolutionary ancient muscle specific MRTF/SRF signalling axis.

Despite the important role of MS1 in skeletal muscle formation and function, little is known about the molecular mechanisms governing its expression. The proximal 1.5 kbp 5'-flanking sequence has recently been shown to be able to direct LacZ expression in adult cardiac and skeletal muscle, with two MEF2 responsive motifs within the proximal flanking sequence essential for the observed cardiac specificity [31]. However, the factors, motifs and regulatory mechanisms governing the skeletal muscle specific expression profile remain unknown. Understanding such mechanisms will give us an exquisite insight into how the MRTF/SRF signalling axis is regulated during myogenesis in addition to expanding our knowledge of the genetic circuits involved in mygenic differentiation.

In this study we investigated the transcriptional regulation of the *ms1 *gene during myogenic differentiation using the C2C12 myoblast cell line as an established model system. We have shown that two myogenic E-Boxes, located within evolutionary conserved regions in the *ms1 *promoter, play distinct roles in recruiting MyoD and subsequently activating the *ms1 *promoter during myogenic differentiation.

## Results

### *Ms1 *transcript is differentially expressed during myogenic differentiation

**Figure 1:**
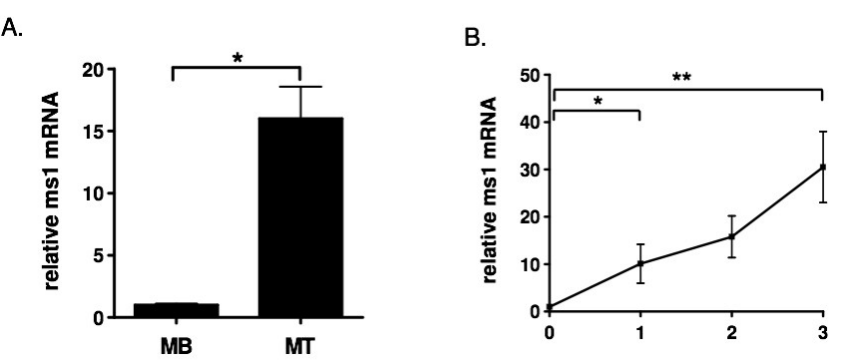
**Quantitative real-time PCR analysis of *ms1 *gene expression during the myogenic differentiation of C2C12 cells**. (A) Total RNA was isolated from C2C12 sub-confluent myoblasts (MB) and myotubes (MT: 3 days post differentiation). The RNA was subjected to reverse transcription followed by quantitative PCR with mouse *ms1 *and EF1α specific primers. (B) Total RNA was isolated from C2C12 cells during myogenic differentiation (day 0 to day 3) and subjected to quantitative PCR. Expression level at day 0 was arbitrarily set at 1. Values are represented as means ± SE of at least three different experiments. (*P < 0.05, **P < 0.05).

*Ms1 *expression is restricted to striated muscle with early developmental expression during myogenesis in both vertebrate and invertebrate models [27,30]. In order to evaluate *ms1 *expression during myoblast differentiation *in vitro*, cultured C2C12 cells, a myoblast cell line established from the leg muscle of the C3H mouse [32], were used. C2C12 is a myoblast cell line, which remains proliferative in the presence of high concentrations of fetal bovine serum. Upon serum depletion, these myoblasts differentiate and fuse with each other into myotubes [33]. Quantitative RT-PCR was conducted using 1 μg of total RNA obtained from subconfluent C2C12 myoblasts (MB) and from C2C12 myotubes differentiated for 3 days (MT). As shown in Figure 1A, there is a significant increase in *ms1 *transcript in differentiated C2C12 cells compared to confluent proliferating C2C12, suggesting a differentiation dependent up-regulation of *ms1 *mRNA.

The temporal expression profile of specific genes expressed during myogenic differentiation can give us an exquisite insight into both the function of the gene and the regulatory processes governing its expression [34,35]. We have therefore measured the temporal expression profile of *ms1 *transcript during the controlled differentiation of C2C12 myoblasts over a three-day period. RT-PCR using RNA isolated on consecutive days during differentiation shows that *ms1 *transcript is significantly induced within the first day of the differentiation process with a maintained increase in expression over the subsequent three days (Figure 1B). *Ms1 *can thus be regarded as an early wave myogenic transcript [36], with this temporal profile having implications for its role and regulation during myogenic differentiation.

### Analysis of the *ms1 *5' upstream DNA sequence

The transcriptional up-regulation of *ms1 *during myogenic differentiation suggests its expression might be targeted by differentiation promoting transcription factors. During myogenesis, differentiation is under the control of E-Box binding myogenic basic helix-loop-helix (MyoD, Myogenin, Myf4 and Myf5) and MADS domain Mef2 proteins [37], with all myogenic genes containing binding motifs for these transcription factors in their promoters and associated regulatory loci. We therefore, by means of comparative sequence analysis, proceeded to analyse the rat *ms1 *5' upstream sequence for enrichment of myogenic transcription factor binding motifs. Using the VISTA software [38] we compared 5 kbp of 5' upstream sequence from rat, with the orthologous locus in human. This compares sequences that shared a common ancestor over 60 million years ago and it has been shown empirically that conserved non-coding sequences, also known as evolutionary conserved regions (ECRs), identified between these species represent ideal candidates as functional transcription factor binding motifs and regulatory domains [39].

**Figure 2:**
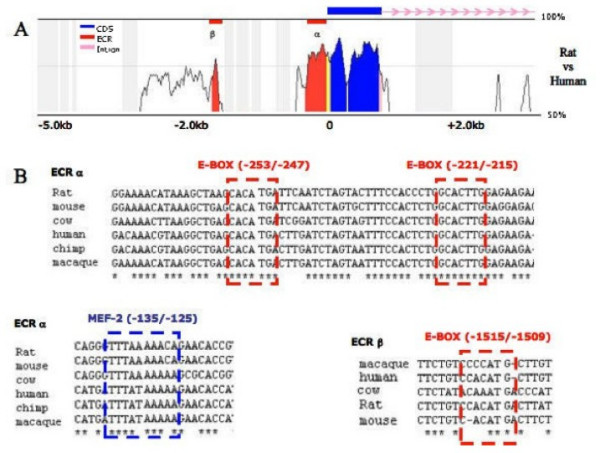
***in silico *analysis of *ms1 *putative promoter**. (A) Phylogenetic comparative analysis of *ms1 *5'-region from *Homo sapiens *and *Rattus norvegicus *(reference sequence), as obtained by VISTA, setting ECR at ≥ 100 bp and conservation at ≥ 80%. (B) Alignment of ECR α and β, containing several putative transcription factor-binding sites, in *Rattus norvegicus, Mus musculus, Bos taurus, Homo sapiens, Pan troglodytes and Macaca mulatta*. Sequences were obtained from the ENSEMBL genome database and aligned using CLUSTAL W.

Our *in silico *analysis (identifying sequences with 80% conservation over a minimum of 100 bp window, Figure 2A) identified two ECRs, α and β (represented in red), within the flanking 5 kbp of 5' upstream sequence. ECR a encompassed the proximal 400 bp upstream of the transcription start site (TSS) and we propose represents the proximal promoter. Within this proximal ECR a putative TATA box (TATT) was found with optimal interspacing from TSS [40], hence suggesting that this constitutes the core promoter. Of particular interest, two E-Box sequences (-253/-247 bp and -221/-215 bp) and a Mef2 motif (-135/-125) were identified within this ECR. Further sequence comparisons, aligning orthologous sequences obtained from ENSEMBL, confirms full sequence conservation of these identified myogenic motifs across multiple species (*Rattus norvegicus, Mus musculus, Bos taurus, Homo sapiens, Pan troglodytes, Macaca mulatta*), supporting a conserved functional role for these motifs (Figure 2B).

In addition to the proximal ECR, a fully conserved E-Box was also identified in the distal ECR β, located 1.5 kbp upstream of the TSS (Figure 2A). This ECR may represent a skeletal muscle enhancer, with such enhancer's common upstream of other myogenic genes including desmin and muscle creatine kinase [41,42]. In summary our comparative analysis suggests that the *cis *hardwiring required for myogenic specific expression is contained within the 2 kbp 5'-upstream sequence with myogenic motifs identified in ECRs α and β.

### Cell specific activity of the *ms1 *promoter

**Figure 3:**
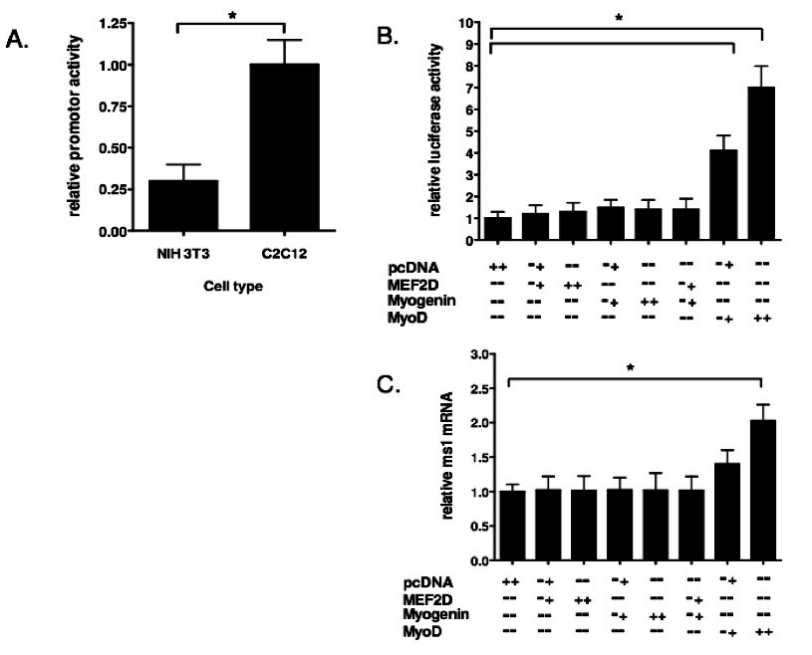
**Analysis of *ms1 *promoter function in myogenic cell lines**. (A) Subconfluent NIH3T3 fibroblasts and C2C12 myoblasts were transiently transfected with a *ms1 *promoter reporter construct spanning from -1585 relative to transcription start site and extending to nucleotide +60. Promoter fragment was cloned into the pGL3-Basic luciferase vector. Activity relative to pGL3-B alone was determined in each cell line 48 hours later, at which point the cells were still subconfluent. The relative activity of the promoter fragment (vs pGL3-B) in each cell line was then determined with relative activity in C2C12 assigned arbitrary value of one (B) Vectors (**+**:0.3 μg, **++**:0.6 μg) expressing Mef2D, myogenin and MyoD were co-transfected in combination with the *ms1 *prompter reporter construct (P-1585/+60) into C2C12 myoblasts as described in (A). Luciferase activity in cells transfected with pcDNA and *ms1 *promoter reporter (P-1585/+60) was arbitrarily set at 1 fold activation. (C) Subconfluent H9c2 myoblasts were transiently transfected with the MRF over-expression vectors (**+**:0.5 μg, **++**:1 μg). 48 hours post transfection, at which point the cells were 80% confluent, total RNA was isolated, reverse transcribed and the expression levels of TATA binding protein (TBP) and *ms1 *were determined by real time PCR. *Ms1 *expression in each sample was normalised to that of TBP. Statistically significant differences are indicated by *P < 0.05.

On the basis of the comparative *in silico *analysis, a 1645 bp fragment of rat genomic DNA (-1585/+60) was obtained by PCR. This DNA fragment, encompassing the α and β ECRs, was sub-cloned into the promoterless pGL3 Basic reporter plasmid and the activity of the resulting P-1585/+60 construct was analysed *in vitro*. The P-1585/+60 wild type promoter reporter (Figure 3A) is approximately four times more active in the C2C12 myoblasts than in the NIH 3T3 mouse fibroblasts. This data suggests that there is sufficient myogenic *cis *information encompassed within the promoter reporter to drive cell-specific activity in a myogenic cellular environment.

### Myogenic factors can modulate the *ms1 *promoter

Since putative E-Box and Mef2 binding motifs are located within ECR α and β, the sensitivity of these motifs to the over-expression of their cognate binding proteins was determined. The rat *ms1 *promoter (P-1585/+60) was transfected into C2C12 myoblasts in the presence or absence of the specific MRFs and Mef2D. In the presence of MyoD, *ms1 *promoter activity was dose dependently increased seven fold compared with control (empty pcDNA3.1 vector). Myogenin alone or in combination with Mef2D, shown to synergistically activate other myogenic promoters [43], did not significantly activate the *ms1 *promoter (Figure 3B). Mef2D alone had no overall activating effect on the *ms1 *promoter.

To determine whether these effects were observed at the endogenous *ms1 *promoter, MRF and Mef2D proteins were ectopically expressed in H9c2 cells, a rat myoblast cell line which can enter the skeletal muscle differentiation programme and expresses an array of skeletal muscle specific contractile and calcium handling proteins [44]. The expression level of endogenous *ms1 *mRNA in both control (empty vector alone) and MRF transfected H9c2 cells was determined by quantitative real-time PCR. MyoD over-expression (1.0 μg) significantly increased *ms1 *mRNA levels by two fold (Figure 3C). Myogenin and Mef2D alone, or in combination, had no effect on endogenous levels of *ms1 *transcript, suggesting MyoD is the primary myogenic activator of the endogenous *ms1 *promoter in a myoblast cellular context. These results suggest that MyoD can target the *ms1 *promoter to enhance its activity, both *in vitro *and *in vivo*.

### Site directed mutagenesis of the *ms1 *promoter

**Figure 4:**
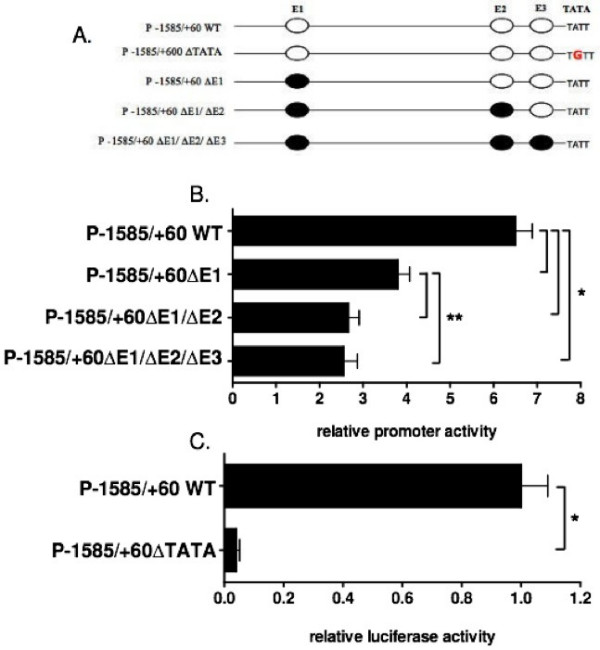
***Ms1 *promoter sensitivity to exogenous MyoD with targeted mutations of putative MyoD binding E-Box sequences**. (A) Schematic representation of the reporter gene constructs used in luciferase assays. Wild type and mutant E-Box sequences are represented in white and black ovals respectively. (B) The E-Box sequences 1,2 and 3 (E1, E2 and E3) contained within the wild type P-1585/+60 construct were subjected to site directed mutagenesis. The subsequent single, double and triple E-Box mutant constructs were transiently co-transfected with MyoD into subconfluent C2C12 myoblasts for luciferase assays that were harvested 48 hours later, when the cells were 80% confluent, Luciferase activity, representing promoter sensitivity to ectopic MyoD expression (fold activation vs pcDNA), was inhibited by approximately 50% in the single E1 mutant with the double E1/E2 mutant resulting in a 67% reduction in activity with respect to wild type promoter sensitivity. Triple E1/E2/E3 mutant maintained the same level of MyoD sensitivity compared to the double E1/E2 mutant. (C) The putative TATA box sequence was subjected to site directed mutagenesis and transiently expressed in C2C12 myoblasts as described above and assayed for luciferase activity. TATA mutation resulted in a 95% reduction in luciferase activity with respect to wild type P-1585/+60 construct. The results are expressed as mean ± SE of at least three different experiments, in triplicate for each construct. Statistically significant differences compared to the appropriate WT construct are indicated by *P < 0.05 and **P < 0.05.

MyoD activates target promoters via heterodimerisation with ubiquitous E2A proteins (E12, E47, E2-5, [45]), which allows a stable DNA binding complex to bind the E-Box sequence (consensus sequence, CANNTG). In order to asses the contribution of the three conserved E-Boxes (in ECR α and β) in mediating MyoD sensitivity, we executed site directed mutagenesis of their binding sites within the *ms1 *promoter reporter construct (Table 1, Figure 4A).

**Table 1:** Oligonucleotide Sequences for PCR and EMSA*

**Cloning PCR**	
P-1585-SacI	5'-TATTCAATGCTTAGTCCTGC-3'
P+60-HindIII	5'-CCAAGCTTCAGGCTACCTGTTTCTTCTC-3'

**Site Directed Mutagenesis PCR**	

ΔT ATA Fw	5'-CACCCTTTCACACCCTGCTTCT**G**TTTAAATCCCAGGCAACTC-3'
ΔT ATA Rv	5 '-GAGTTGCCTGGGATTTAAA**C**AGAAGCAGGGTGTGAAAGGGTG-3'
ΔE1 Fw	5 '-CACTGAACAGGTGCTGTTTCTCTGTC**GTTAAG**ACTTATCCTTTCAG TTCTCTTAAAA-3'
ΔE1 Rv	5 '-TTTTAAGAGAACTGAAAGGATAAGT**CTTAAC**GACAGAGAAACAGCA CCTGTTCAGTG-3'
ΔE2 Fw	5'-CTTTCCACCCTGGC**G**C**GG**GGAGAAGAAAGGAG-3'
ΔE2 Rv	5'-CTCCTTTCTTCTCC**CC**G**C**GCCAGGGTGGAAAG-3'
ΔE3 Fw	5 '-CAAGGAAAACATAAAGCTAAGC**G**C**GG**GATTCAATCTAGTACTTC-3'
ΔE3 Rv	5 '-GAAGTACTAGATTGAATC**CC**G**C**GCTTAGCTTTATGTTTTCCTTG-3'

**Quantitative PCR**	

MS1 Fw	5'-GTGACAGCATAGACACAGAGGAC-3'
MS1 Rv	5'-CACTGCTGCCCACCTGCCTT-3'
EF1-α Fw	5'-AGCTTCTCTGACTACCCTCCACTT-3'
EF1-α Rv	5'-GACCGTTCTTCCACCACTGATT-3'

**ChIP Quantitative PCR**	

E1-1526	5'-CACATTTTTATCTGGTCTAATACACTG-3'
E1-1482	5'-ATTTTTAATAGAACTGAAAAGAGAAGTCA-3'
E2-281	5'-TAAGGTCAAGGAAAACATAAAGCTA-3'
E2-190	5'-ACGGATATGTTCCCTCCTCTCTC-3'

**EMSA**	

E-Consensus	5'-CCCTTGGAACATCTGTCGATGCTG-3'
E1-MS1	5'-TTCTCTGTCCACATGACTTATCCT-3'
E2-MS1	5'-ACCCTGGCACTTGGAGAAGAA-3'
E3-MS1	5'-AGCTAAGCACATGATTCAATC-3'

*Mutated oligonucleotides are indicated in bold and underlined.

Reporter constructs containing mutated sequences for the three E-Boxes, singular and in combination (Figure 4A), were co-transfected with the MyoD over-expression plasmid into C2C12 myoblasts. Mutating E1 (P-1585/+60ΔE1) reduced promoter sensitivity to ectopic MyoD expression by 50% (from 7- to 3.5-fold; Figure 4B), suggesting this E-Box (located in ECR β) is important for promoter sensitivity to MyoD. The additional mutation in E2 (P-1585/+60ΔE1/ΔE2) further attenuated promoter sensitivity to 2.5 fold (~67% decrease) (Figure 4B). The combined triple E-Box mutant (P-1585/+60ΔE1/ΔE2/ΔE3) did not result in a further decrease in promoter sensitivity to MyoD compared to the double mutant (Figure 4B). These results suggest that both E1 and E2, but not E3, are required for MyoD activation of the rat *ms1 *promoter.

The putative TATA box (TATT) was also mutated exchanging the adenine, at second position for a guanine (TATT to TGTT). This resulted in a dramatic 95% decrease in promoter activity (Figure 4C) in C2C12 myoblasts. Comparable loss of activity was observed in H9c2 myoblasts and NIH 3T3 fibroblasts (data not shown) suggesting loss of activity is not cell type specific. This suggests that this proximal TATT sequence represents a *bona fide *TATA box.

### *In vitro *binding of MyoD at distal and proximal E-Box sequences

**Figure 5:**
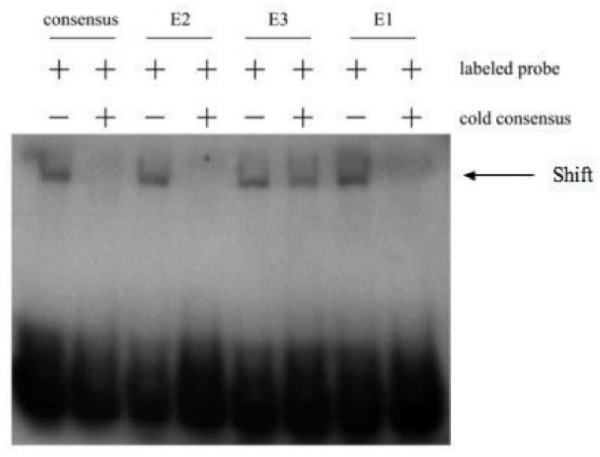
**EMSA analysis of E1, E2 and E3 in the ms1 promoter**. DIG-labelled oligonucleotide probes for the MyoD E-Box binding consensus, E1, E2 and E3 binding sites were incubated with whole cell protein extracts made from subconfluent C2C12 myoblasts. Competition experiments were performed using a 200-fold excess of unlabeled MyoD E-Box consensus probe. Arrow indicates the resulting bandshifts.

The present mutagenesis analysis suggests that E1 and E2, but not E3, play an important role in mediating MyoD sensitivity to the rat *ms1 *promoter. To further elucidate the biological importance of the E1 and E2 sequences, we synthesised specific oligonucleotides (Table 1) containing the E-Box elements present in ECR α and β, E1, E2 and E3. These digoxigenin (DIG)-labelled double-stranded oligonucleotides were then incubated with a cold MyoD binding consensus E-Box sequence control in EMSA experiments with whole cell extracts from C2C12 myoblasts. As shown in Figure 5, incubation of C2C12 whole cell extracts with both MyoD consensus and E1, E2 and E3 sequences results in a specific DNA-Protein band shift.

The E1 and E2 shifted bands were successfully competed with excess unlabelled MyoD consensus sequence, suggesting these probes were bound by MyoD protein. In contrast, E3 could not be competed with unlabelled excess of MyoD consensus suggesting other E-Box binding proteins are shifting E3 *in vitro*. All three E-Box shifted bands were competed with unlabelled excess of self, confirming shifted bands were specific to each sequence (data not shown).

In agreement with our mutagenesis analysis, our EMSA data suggests that MyoD can target E1 and E2, but not E3. Other E-Box binding proteins expressed in C2C12 myoblasts are able to bind E3. Future experiments are aimed at determining their identity.

### Direct binding of MyoD to the endogenous ECRs within the *ms1 *promoter

Our results suggest that the E1 and E2 sites are essential for *ms1 *reporter gene function. We then utilised chromatin immunoprecipitation (ChIP) to determine whether MyoD is physically recruited to the endogenous ECRs *in vivo*, and determine the temporal dynamics of MyoD recruitment during C2C12 differentiation.

**Figure 6:**
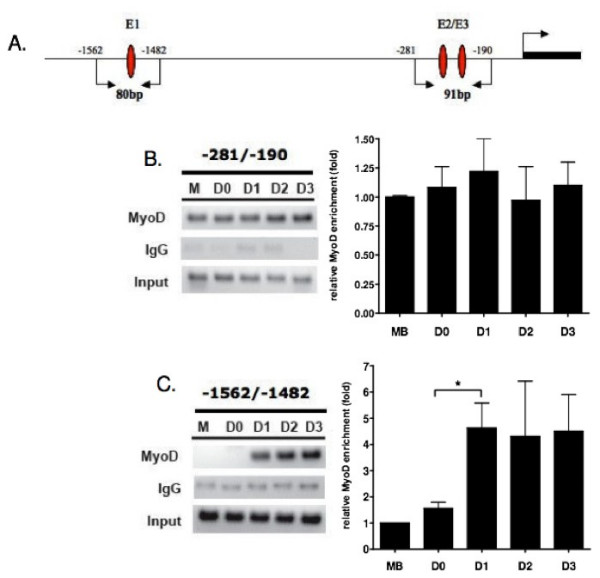
**Chromatin immunoprecipitation assay performed on differentiating C2C12 cells to evaluate in vivo MyoD binding at the *ms1 *promoter**. (A) A schematic map of the amplified DNA fragments (product size) and the primer locations encompassing E1, E2 and E3. TSS position is also illustrated. Proteins were cross-linked to the DNA (in C2C12 cells during myogenic differentiation) with formaldehyde, DNA was sheared by sonication, and Abs directed against IgG or MyoD were added to precipitate any protein-DNA complexes. The precipitated complexes were pre-cleared with protein A beads. Samples analyzed included proliferating, subconfluent myoblasts (M), confluent myoblasts harvested prior to the induction of differentiation (D0), myoblasts subjected to differentiation conditions for 24 hours (D1), and differentiating myotubes at 48 hours (D2) or 72 hours (D3) post-differentiation. Quantitative real time PCR were performed on isolated DNA using primers encompassing the proximal (B) and distal (C) E-Box sequences (E2/E3 and E1 respectively). Amplification was quantified and normalised to the input of each sample. The results are expressed as mean ± SE of at least three different ChIPs. Statistically significant differences in fold enrichment are indicated by *P < 0.05. Representative PCR reactions were stopped in the linear amplification range and run on agarose gel for visualisation.

An ECR-specific quantitative PCR (Figure 6A) was performed on formaldehyde-crosslinked, sheared chromatin isolated during C2C12 differentiation, which was immunoprecipitated with MyoD and IgG specific antibodies. As shown in Figure 6B, MyoD appears to be constitutively bound at the E2 domain during differentiation, thus MyoD binding precedes the induction of *ms1 *transcript (Figure 1B). Interestingly MyoD is not bound to E1 until day 1 (Fig. 6C), which coincides with transcriptional induction of *ms1 *during the differentiation process. A five-fold enrichment in relative binding of MyoD is present at day 1 compared to day 0, with this level of enrichment maintained at E1 during the subsequent three days. These data suggest that MyoD targets both the E1 and E2 domains *in vivo *during C2C12 differentiation. However, temporal binding at E1 coincides with differentiation dependent transcriptional induction. We speculate that the ECR β represents a differentiation-dependent skeletal muscle enhancer, with temporal binding required for differentiation-dependent transcriptional induction of the *ms1 *promoter.

## Discussion

Understanding the mechanisms through which SRF activity is regulated during myogenesis is important if we want to expand our knowledge of the gene regulatory pathways and networks that drive skeletal muscle determination and differentiation. Targeting and activation of muscle specific genes by SRF is dependent on specific association with the powerful co-activators, MRFT-A and -B, and Rho-A signalling. We, and others, have recently shown the actin binding protein, MS1 (STARS), to be both required and sufficient for muscle specific activation of the RhoA/MRTF/SRF signalling axis. Therefore, understanding the transcriptional regulatory mechanisms governing *ms1 *expression will give us a key insight into how the MRTF/SRF axis is regulated during myogenic differentiation.

As a first step towards understanding the transcriptional mechanisms governing *ms1 *expression in muscle differentiation, we analysed *ms1 *expression in differentiating C2C12 cells. A robust induction in *ms1 *expression was observed during the first day of differentiation suggesting *ms1 *is an early 'wave' myogenic transcript [34]. Fernandez and colleagues [48] have reported that C2.7 myoblast fusion and differentiation is dependent on SRF, as a consequence of its role in regulating MyoD expression. In addition, MyoD and SRF have been shown to physically interact and syngerstically activate target promoters, with consensus SREs enriched in *bona fide *MyoD target promoters [35]. Considering this, one would expect SRF activity to coincide with MyoD during early myogenic differentiation. We propose this early expression of *ms1 *drives muscle specific activation of the MRTF/SRF axis, coupling this pathway with MyoD expression and activity.

An *in silico *comparative sequence analysis suggested that the proximal 1.5 kbp 5'-upstream sequence would be capable of driving muscle specific transcription. Within this region, two evolutionary conserved regions were identified, both of which were enriched with ultra conserved binding motifs for key myogenic regulatory factors. The proximal ECR also contained a conserved TATA box located at the correct distance from transcription start site suggesting it constitutes the core promoter. This 1.5 kbp promoter fragment was significantly more active in a muscle versus a non-muscle cell type (Figure 3A) supporting the *in silico *derived hypothesis. It is of interest that in a recent study the proximal 1.5 kbp 5'-upstream region of the mouse STARS gene was able to direct transgenic lacZ expression in adult skeletal muscle *in vivo*, thus supporting our *in vitro *data [31].

We speculated that the ultra conserved myogenic regulatory motifs encompassed within this promoter fragment would be important for muscle specific activity. A significant increase in promoter reporter activity was observed with the ectopic expression of the MRF, MyoD. However myogenin and Mef2D were not able to activate the *ms1 *promoter reporter in this myoblast cellular environment. This pattern of sensitivity was also the same at the level of endogenous *ms1 *transcription.

The observed specific sensitivity to MyoD complements the endogenous *ms1 *expression profile during C2C12 differentiation. MyoD is responsible for myogenic gene activation during early stages of differentiation [2,35], the time at which *ms1 *transcription is induced. Myogenin and Mef2 proteins are themselves subsequently induced by MyoD, and in a combinatorial manner drive expression of late myogenic genes as well as consolidating and maintaining expression of early myogenic genes [43,49]. In addition this specific sensitivity may suggest that only MyoD and not myogenin can target the myogenic E-Boxes within the *ms1 *promoter in a myoblast cellular environment. This is not uncommon, for example, MyoD targets myogenic E-Boxes within the chicken MLC-1 promoter, which cannot be bound by myogenin [50].

It is of interest that Mef2C binding to the Mef2 consensus sequence identified in our comparative analysis (ECR α) has been shown to mediate basal and stress inducible cardiac specific promoter activity both in *in vitro *and *in vivo *(Kuwahara et al, 2007). In addition, skeletal muscle specific deletion of the Mef2C isoform at early, but not late times of embryogenesis, results in mice with disorganised myofibers that are perinatal lethal (Potthoff et al, 2007). Our data (Fig. 3) suggest that this Mef2 motif in the *ms1 *promoter is not required for the initial MyoD mediated induciton of ms1. However, our data do not preclude the possibility that Mef2D may contribute to ms1 expression later in myogenesis or that other isoforms of Mef2 may contribute to ms1 activation.

We hypothesised that the observed MyoD sensitivity was via specific targeting of MyoD to the ultra conserved E-Box's, E1, E2 and E3, located within the two evolutionary conserved regions. Indeed, via site directed mutagenesis and electromobility shift assays, we demonstrated that E1 and E2, but not E3 appear to be targeted by MyoD. Surprisingly a significant level of MyoD sensitivity was retained in the triple E-Box mutant. This may suggest the presence of other non-conserved E-Boxes targeted by MyoD within the promoter reporter or alternatively be a result of the up-regulation of other myogenic transcription factors by MyoD, which subsequently target and activate the *ms1 *promoter at other motifs. It is of interest to note the presence of a conserved SRE within the proximal ECR, which can be bound by SRF in cardiomyocytes *in vivo *(Ounzain S, unpublished). MyoD upregulation is predicted to increase the activity of SRF [48], so it is possible for SRF to target and increase activity of *ms1 *promoter independent of MyoD binding at E1 and E2. It is also interesting to consider the up-regulation of the muscle specific isoform of Mef2D (Mef2D1b), which we suspect may target the *ms1 *promoter in late stages of differentiation.

Many muscle specific genes are activated at different times during the myogenic differentiation process. Numerous studies suggest that this differential expression of each target gene is a product of specific temporal binding of MyoD at distinct E-Boxes within the *cis *regulatory domains of the gene, which itself is coupled to chromatain modification and remodelling [3]. We therefore used ChIP to measure *in vivo *binding of MyoD at the *ms1 *E1 and E2 domains during C2C12 differentiation. Our analysis shows that MyoD is constitutively bound at the proximal E2 domain during differentiation, with binding preceding the induction of *ms1 *transcript at day 1 (Figure 6B). MyoD is capable of binding target sequences prior to gene activation, acting in a repressive manner via the specific recruitment of repressive chromatin remodelling complexes. Interestingly, *ms1 *transcriptional induction at day 1 (Figure 1B) coincides with MyoD binding at E1, located within the ECRβ (Figure 6C). This suggests MyoD targeting at E1 is required for transcriptional activation of *ms1 *and we therefore propose that ECRβ could represent a differentiation-dependent skeletal muscle enhancer.

Prior to activating transcription, MyoD associated with HDACs serves to mark myogenic genes for subsequent differentiation cues and thus activation [51]. It is conceivable that MyoD binding at the *ms1 *promoter prior to differentiation primes the promoter in a 'poised' myogenic state. The recruitment of HDACs by MyoD causes the local chromatin environment to be compacted and will prevent the association of MyoD and other activating factors with specific cognate binding sites. However appropriate differentiation signals can stimulate MyoD to toggle between HDAC and HAT recruitment in addition to association with differentiation-specific myogenic factors [3]. This facilitates binding of MyoD with other E-Boxes (E1) and allows the formation of activating transcriptional complexes.

MyoD acetylation has recently been implicated as a central mediator of the temporal activation of muscle specific gene expression during myogenesis [47]. We therefore cannot rule out the possibility that during C2C12 differentiation an acetylated MyoD form with increased DNA binding efficiency [52] is capable of binding the E1 domain, resulting in the temporal activation of *ms1 *transcription. It is of interest that preliminary data indicate that trichostatin A, a HDAC inhibitor, is able to increase ms1 transcript abundance in H9c2 myoblasts (Ounzain S, unpublished).

Taking the present data together, we propose a model whereby MyoD binding at the *ms1 *proximal ECR in myoblasts represses *ms1 *transcription via the recruitment of HDACs. This proximal binding is essentially priming the *ms1 *promoter, placing it in a poised state for sensing appropriate differentiation cues. Upon differentiation MyoD associates with HATs and SWI/SNF, which subsequently causes remodelling of the local chromatin environment, allowing MyoD to bind E1 within the distal ECR or alternatively, temporally acetylated MyoD binds the E1 domain. Further targeting of HATs and specifically SWI/SNF complexes (at E1) can facilitate binding of TBP and other factors involved in polymerase II pre-initiation complex formation and promote transcriptional elongation [53-58]. We thus speculate that temporal targeting of MyoD at E1 is required to establish the optimum environment for Pol II action and robust transcription.

In summary our data suggests that MS1 is a key component of a MyoD generated feed-forward regulatory circuit, where factors induced by MyoD (like *ms1*) feed-forward to regulate late MyoD activity (via SRF) at subsequent target genes, therefore acting to temporally pattern the timing of gene expression during skeletal myogenesis. This MyoD-MS1-SRF feed-forward network would serve to consolidate and amplify the myogenic cascade. Indeed SRF itself acts in combination with MyoD to activate many downstream genes, thus, through the specific regulation of *ms1*, MyoD is able to synchronize SRF activity with its own and thus collaborate to mediate the temporal activation of downstream genes.

We believe this is the first study to demonstrate a direct link between MyoD activity and SRF transcriptional signalling, with *ms1 *serving as the nodal point to integrate these two central myogenic regulatory networks. It is of interest that in cardiomyocytes MS1 serves a similar function in that it integrates the Mef2 and SRF signalling networks, providing a link for crosstalk between them [31]. This is thus a conserved emerging paradigm for MS1 function both in cardiac and skeletal muscle. In addition we have data to suggest that MS1 is capable of integrating the GATA4 cardiogenic network with SRF activity [59].

This study also has implications for myogenic disease phenoptypes. IGF-1 and IL-4, both central mediators of post-natal skeletal muscle regeneration are regulated by SRF in response to stress [60]. Therefore understanding the molecular mechanisms regulating *ms1 *expression may allow us to identify and develop therapeutic strategies for the up-regulation of *ms1 *gene expression in a disease phenotype, which would facilitate regeneration via stimulation of SRF activity and resulting up-regulation of IL-4 and IGF-1.

## Conclusion

Identification of direct transcriptional targets of MyoD and de-convolution of the transcriptional regulatory networks that operate in muscle cells represent an essential target if we are to understand not only how muscle differentiates but also how it responds to stress and damage, therefore allowing regeneration. We have demonstrated that via temporal binding of MyoD at distinct E-Boxes within the *ms1 *promoter, *ms1 *potentially serves to integrate the MyoD and SRF myogenic regulatory circuits, thus driving a feed-forward auto-regulatory circuit that consolidates and amplifies the myogenic phenotype. We believe this is the first study to describe a direct link between MyoD activity and SRF signalling, with *ms1 *allowing cross talk to occur between these two independent myogenic networks. This implicates MS1 as a key factor involved in myogenic differentiation and potentially regeneration.

## Methods

### Cell cultures

The H9c2 rat myoblast and NIH3T3 mouse fibroblast cell lines were grown in a humidified atmosphere containing 5% CO_2 _at 37°C in DMEM supplemented with 10% FCS, 2 mM glutamine, streptomycin and penicillin (each at 10 g/liter). C2C12 mouse skeletal myoblasts were cultured in DMEM supplemented with 20% FBS, 100 units/ml penicillin and 100 ug/ml streptomycin and maintained at 37°C in 5% CO2. The differentiation of C2C12 myoblasts into myotubes was achieved by the addition of differentiation medium (DMEM supplemented with 2% horse serum) at confluence for up to 4 days with medium change every 2 days.

### Plasmid constructs

The rat MS1 gene sequence was obtained from GenBank (NC_005106) and was used to design primers that would amplify the 5' flanking region. Two oligonucleotides, P-1585-SacI and P+60-HindIII (Table 1), were designed to amplify a portion of DNA sequence starting -1585 bp upstream of the transcription start site (+1), with primers tailed with restriction sites for *SacI *and *HindIII *restriction enzymes respectfully. In addition to template (rat genomic DNA, WKY strain) and primers P-1585-SacI and P+60-HindIII, the reaction contained 0.2 mM dNTPs, Expand polymerase buffer and 5 units of *Taq *Expand high fidelity polymerase (Roche). Reaction was subjected to 35 cycles of amplification (45 s at 94°C, 45 s at 59°C and 90 s at 72°C). The PCR product was cloned into the vector pGEM-T Easy (Promega) and sequenced to ensure fidelity of amplification. The verified plasmid was then cut with SacI/HindIII and the released -1585 promoter fragment was purified and cloned into the pGL3-Basic (Promega) reporter vector, *SacI/HindIII *digested. The subsequent construct was designated P-1585/+60WT. The MyoD and Myogenin expression plasmids were provided by Dr Andrew Lassar [62,63] and the Mef2D expression plasmid was a gift from Dr Eric Olson (University of Texas, South Western Medical centre) [64].

### Site directed mutagenesis

The P-1585/+60WT construct was subjected to site directed mutagenesis to mutate the TATA, E-Box1, 2 and 3 binding sites, using the Quik Change II site-directed mutagenesis kit (Stratagene). The mutagenic primers used to generate the TATA and E-Box mutations are reported in Table 1. Site-directed mutagenesis was performed according to the manufacturers instructions. Mutated fragments were then re-cloned into the Sac I/Hind III sites of the corresponding pGL3-Basic vectors. Double (ΔE1/ΔE2) and triple-site (ΔE1/ΔE2/ΔE3) mutation constructs, were generated by consecutive rounds of mutagenesis. The resulting plasmids were confirmed by DNA sequencing.

### Transient transfections and luciferase assays

C2C12 and NIH 3T3 cells were transfected using the cationic transfection reagent, Jet Pei (QBiogene), according to the manufacturers protocol. Cells were seeded in six well plates. Twenty-four h post plating; the cells were co-transfected with 0.5 μg of promoter-luciferase construct and equimolar amounts for the other plasmids used (total of 0.6 μg). The total amount of DNA was kept constant using empty vector (pcDNA3.1). To normalise for transfection efficiency, the pRL-TK (Promega) expression plasmid containing *Renilla *luciferase (20 ng per well) was co-transfected. *Firefly *and *Renilla *luciferase activities were measured at 48 h post-transfection using the Dual-Glo™ Luciferase assay system (Promega) and a Lumat LB9507 luminometer (Berthold Technologies). All plasmids were purified using Qiagen columns (Qiagen) and at least two preparations per plasmid were tested. The transfection efficiency was normalised using the *Renilla *luciferase activity levels and each transfection was performed in triplicate and repeated in a minimum of three independent experiments.

### RNA isolation and quantitative real time RT-PCR

H9c2 cells seeded in six well plates were transfected with up to 1.5 μg of expression plasmid using JetPei (as above). Total amount of DNA was kept constant using empty vector (pcDNA3.1). Forty-eight h post transfection or 24 h with TSA, total RNA was isolated from cells using the RNeasy Mini Kit (Qiagen) according to the manufacturer's instructions. RNA (1 μg) was reverse transcribed to cDNA using oligo (dT) and Superscript II Reverse Transcriptase (Invitrogen). Rat *ms1 *mRNA expression was analysed using quantitative PCR with fluorescent-labelled TaqMan probes (Rat *ms1 *primers and probe, Cat No. Rn00598518_m1, Applied Biosystems). TBP was used as the internal control (Cat No. Mm00446973_m1, Applied Biosystems). PCR amplifications were performed in duplicate in 25 μl containing 2 μl cDNA template in 2× PCR master mix (Applied Biosystems). Amplification conditions as follows: 50°C, 2 min; 95°C, 10 min; 40 cycles of 95°C for 15 s followed by 60°C for 1 min. Reactions were performed and products detected using an ABI-Prism HT 7900 sequence detector (Applied Biosystems). The level of expression of *ms1 *mRNA was normalised to TBP expression. For C2C12 myoblasts, cells were cultured and harvested for RNA extraction according to the time course of differentiation. Total RNA was isolated using Trizol reagent (Invitrogen) according to the manufacturers instructions. Total RNA (2 μg) was reverse transcribed with Superscript III (Invitrogen). Quantitative PCR was performed with Qiagen HotStart Taq Master Mix and SYBR Green I as described previously (Schmittgen and Zakrajsek, 2000) using primers for *ms1 *and EF1-α (Table 1). Amplifications were performed in a DNA Engine Opticon System (MJ Research) and quantified. *Ms1 *mRNA levels were normalised to EF1-α mRNA levels.

### Electromobility shift assays (EMSA)

Whole cell extracts were prepared from cultured C2C12 myoblasts using the CelLytic™-M cell lysis extraction reagent (Sigma) according to the manufacturers instructions. The protein concentrations were determined using the Bradford assay (Bio Rad). Whole cell extracts were incubated for 15 min with 0.5 pmol of the appropriate digoxigenin (DIG)-labelled double-stranded oligonucleotide probe (Table 1) in a 20 μl reaction containing binding buffer (Roche), 1 μg poly (dI-dC) (Roche) and 0.1 μg poly L-lysine (Roche). The probes were end-labelled with DIG-11-ddUTP (Roche). The reaction was stopped by the addition of 5 μl loading dye (Roche). For competition experiments, a 200-fold excess of unlabeled double-stranded oligonucleotide (Table 1) was added to the reaction. The samples were then run on a 4% non-denaturing polyacrylamide gel using 1× TGE buffer (50 mM Tris, 380 mM Glycine and 2 mM EDTA), and transferred to a positively charged nylon membrane (Amersham Biosciences). DIG-labelled oligonucleotides were visualised by incubation with alkaline phosphatase-labelled F(ab)_2 _anti-DIG Ab, followed by chemiluminescence reaction with 100 μg/ml CPSD substrate (Roche).

### Chromatin immunoprecipitation assay (ChIP)

C2C12 ChIPs were performed using MyoD (de la Serna et al 2005) and IgG (Santa Cruz) antibodies. The ChIP was performed as described previously [57], except that immune complexes were eluted with 0.1 M NaHCO3 and 1% SDS, and following reversal of cross-links, the eluate was digested by proteinase K digestion and purified using the Qiagen PCR Purification kit (28106). 4 μl of DNA eluted from the column was used for PCR. Inputs consisted of 1% chromatin before immunoprecipitation. PCRs were performed with Qiagen HotStart Taq Master Mix using primer sets reported in Table 1. Amplification was quantified by a DNA Engine Opticon System (MJ Research), then normalised to the input of each sample. PCR reactions run on agarose gels were stopped in the linear range and visualised using SYBR Green I.

### Comparative DNA analysis

Comparative sequence analysis of human and rat was performed using web-based software available at the Lawrence Berkley Laboratory genome website (VISTA). Orthologous sequences from *Mus musculus, Bos taurus, Homo sapiens, Pan troglodytes and Macaca mulatta *were obtained from the ENSEMBL genome data base and aligned using CLUSTAL W.

### Data Analysis

Data are expressed as means ± SE (represented as error bars). Comparisons were made using the Student's *t *test, considering P < 0.05 statistically significant.

## Abbreviations

MRF: myogenic regulatory factor; SRF: serum response factor; MRTF: myocardin related transcription factor; STARS: striated muscle activator of Rho signalling; MEF2: myogenic enhancer factor 2; ECR: evolutionary conserved region; TSS: transcription start site; DIG: digoxigenin; EMSA: electromobility shift assay; ChIP: chromatin immunoprecipitation assay; TSA: trichostatin A; HDAC: histone deacetylase; HAT: histone acetyltransferease; SRE: serum response element; MLC-1: myosin light chain 1; MS1: myocyte stress 1; IGF-1: insulin growth factor 1; IL-4: interleukin 4; DMEM: dulbecco's modified eagle medium; FCS: fetal calf serum; TBP: TATA binding protein; TGE: tris glycine EDTA; EDTA: ethylenediaminetetraacetic acid; SDS: sodium dodecyl sulfate.

## Authors' contributions

SO planned the study, carried out the experimentation and drafted the manuscript with input from CSD, ANI and NWC. CSD performed RT-PCR and ChIP on C2C12 cells as well as contributing to the analysis and interpretation of the data. NJS participated in the co-ordination of the study. ANI contributed to the planning and co-ordination of the study in addition to analysis and interpretation of the data. NWC supervised the study and participated in drafting of the manuscript. All authors read and approved the final manuscript.
